# Microphotochemistry: 4,4'-Dimethoxybenzophenone mediated photodecarboxylation reactions involving phthalimides

**DOI:** 10.3762/bjoc.7.121

**Published:** 2011-08-02

**Authors:** Oksana Shvydkiv, Kieran Nolan, Michael Oelgemöller

**Affiliations:** 1 School of Chemical Sciences, Dublin City University, Dublin 9, Ireland; 2 School of Pharmacy and Molecular Sciences, James Cook University, Townsville, QLD 4811, Australia

**Keywords:** microflow, microreactor, photochemistry, photodecarboxylation, phthalimide

## Abstract

A series of 4,4'-dimethoxybenzophenone mediated intra- and intermolecular photodecarboxylation reactions involving phthalimides have been examined under microflow conditions. Conversion rates, isolated yields and chemoselectivities were compared to analogous reactions in a batch photoreactor. In all cases investigated, the microreactions gave superior results thus proving the superiority of microphotochemistry over conventional technologies.

## Introduction

**Scheme 1:**
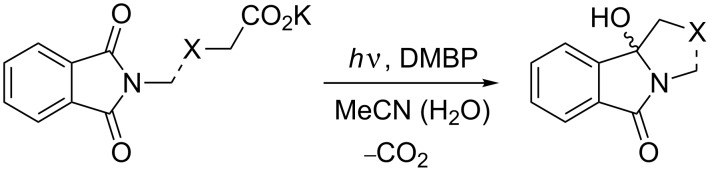
General photodecarboxylation involving phthalimides (the broken line indicates intra- as well as intermolecular reactions).

Organic photochemistry is a highly successful synthesis method that allows the construction of complex molecules with a “flick of a switch” [1–4]. Light is furthermore considered a clean “reagent” and consequently, photochemistry has contributed extensively to the growing field of Green Chemistry [5–7]. It is therefore surprising that synthetic organic photochemistry has been widely neglected by the chemical industry. In fact, most photochemical production processes in industry were developed and realized decades ago [8–11]. A major drawback of photochemistry as a modern research and development (R&D) tool has been the usage of specialized reactors and lamps, which are often considered “exotic” by synthetic chemists [12]. Over the last decade, microflow chemistry has emerged as a new tool in preparative organic chemistry [13–16]. Microflow reactors (μ-reactors) offer a number of advantages for photochemical transformations. In particular, their narrow reaction channels enable extensive penetration of light even at high chromophore concentrations. In addition, products are removed from the irradiated area thus preventing light-induced follow-up reactions or decompositions [17–19]. Recently, a number of photoreactions in microreactors have therefore been described [20–23] and specialized micro-photoreactors for laboratory- to technical-scale synthesis have been developed [24–26]. We have recently reported on acetone-sensitized photodecarboxylation (PDC) reactions of phthalimides in a commercially available microreactor [27]. The photochemistry of phthalimides and its analogues has been intensively studied over the last decades [28–32]. Among the various transformations developed, photodecarboxylation reactions have emerged as efficient and powerful alkylation procedures with high quantum yields of up to 60% [33–34]. Selected transformations have also been realized on a semi-technical scale using an advanced falling-film batch reactor equipped with a 308 nm excimer light source [35–36]. However, the established PDC protocol utilizes UVB light for the activation step (direct or acetone sensitized), thus limiting the desired future application of LEDs [37]. We have therefore investigated the usage of 4,4’-dimethoxybenzophenone (DMBP) as a photocatalyst that absorbs readily in the UVA region. In this publication we present preliminary results of five DMBP mediated model transformations (Scheme 1). All reactions were previously studied under acetone-sensitized conditions using UVB light [27].

## Results and Discussion

### Experimental setups

**Figure 1:**
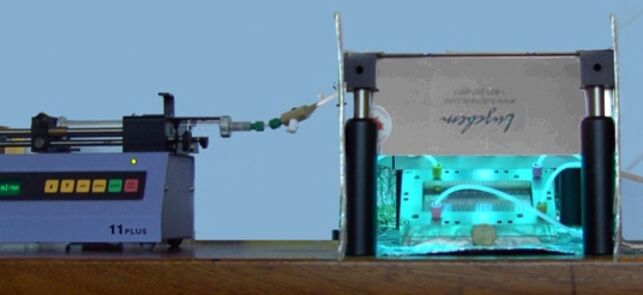
Microreactor (dwell device, mikroglas chemtech) under a UV exposure panel (Luzchem) and connected to a syringe pump.

The reaction setup is shown in Figure 1. A commercially available dwell device (mikroglas chemtech) was placed under a UV panel (Luzchem) fitted with five 8 W UVA lamps (λ = 350 ± 25 nm). The reactor itself was fabricated from Foturan™ glass, which has a transmission of approximately 30% at 300 nm, and consisted of a heat-exchanging channel on the top and a serpentine reaction channel on the bottom. The reaction channel had a total path length of 1.15 m with 20 turns, a depth of 0.5 mm, a width of 2 mm and a total volume of 1.68 mL. The reaction mixture was loaded into a programmable syringe pump, degassed with nitrogen, pumped through the microreactor (flow rate: 0.028 mL/min) and collected in a flask outside the irradiated area. In a parallel series of experiments, a conventional Rayonet chamber reactor (RPR-200) equipped with sixteen 8 W UVA lamps in a circular arrangement was used for batch reactions. A Pyrex Schlenk flask, with a transmission of approximately 30% at 300 nm, of 32 mm inner diameter and equipped with a cold finger of 24 mm diameter, thus creating an effective path length of 4 mm, was inserted into the chamber. After a fixed irradiation time of 1 h, which was not optimized, and work-up the crude reaction products were analyzed by ^1^H NMR spectroscopy and conversions and selectivities were determined. In represented cases the pure products were isolated for characterization purposes from the batch processes. Due to the small amounts used under microflow conditions, purification and isolation of products was not attempted. Previous work has, however, demonstrated that isolated yields typically match conversion rates [38].

### Wavelength matching and light penetration

**Figure 2:**
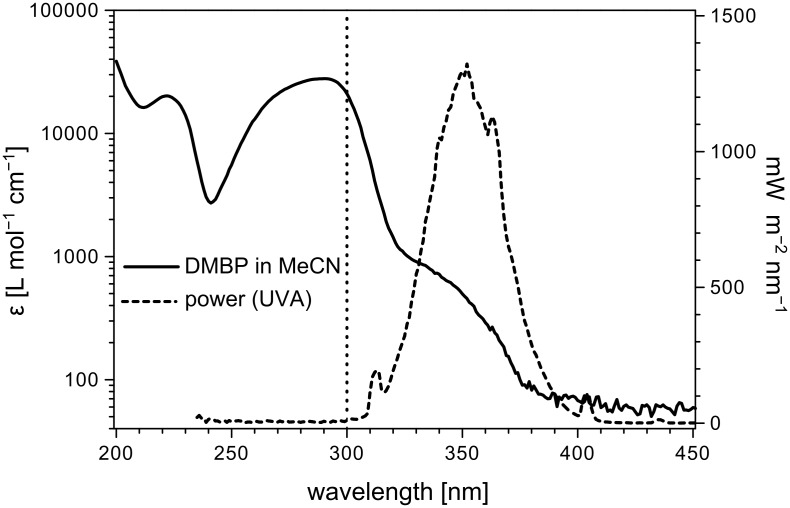
UV-spectrum of DMBP (in MeCN) versus emission spectrum of the UVA lamp. The vertical dotted line represents the cut-off wavelength of Foturan™ and Pyrex at 300 nm (approx. 30% transmission).

When the absorption spectrum of DMBP in acetonitrile was compared to the emission spectrum of the chosen UVA lamp (Figure 2), its important n→π* absorption matched well with the emission maximum of the light source. At 350 nm, an extinction coefficient (ε_350 nm_) for DMBP of 496 L mol^−1^ cm^−1^ was determined. In contrast, the crucial n→π* absorption maximum of *N*-alkylated phthalimides in acetonitrile lies around 290 nm and consequently, photoprocesses induced by direct excitation may be neglected [39].

**Figure 3:**
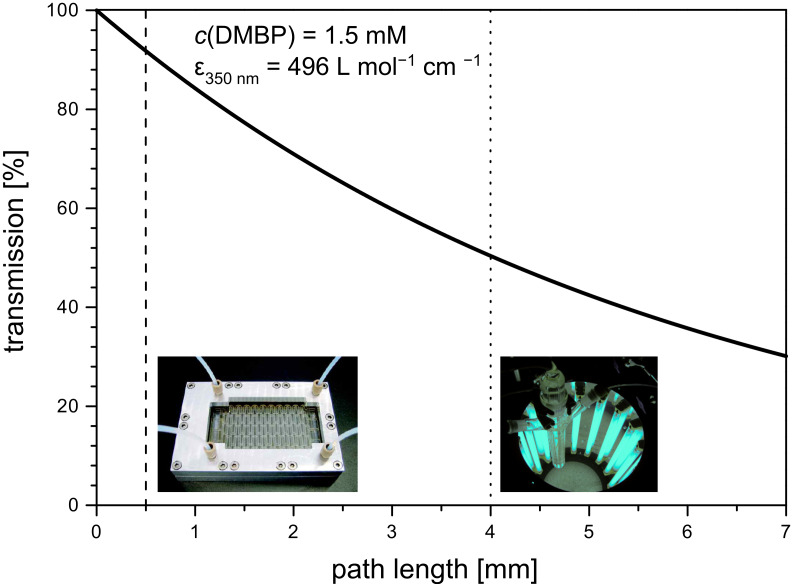
Light-penetration profile for a 1.5 mM solution of DMBP at 350 nm. The vertical lines represent the path length in the dwell device (vertical dashed line) versus the effective path length in the Schlenk flask (vertical dotted line).

The light transmission for a 1.5 mM DMBP solution in acetonitrile (Figure 3) was subsequently calculated using the Beer–Lambert law [8]. As indicated by vertical lines, both set-ups guaranteed complete penetration of light at 350 nm. As would be expected from its much smaller path length, the light transmission in the microchannel was superior at 92%, compared to 50% in the batch system.

### α-Photodecarboxylation of *N*-phthaloylglycine

**Scheme 2:**
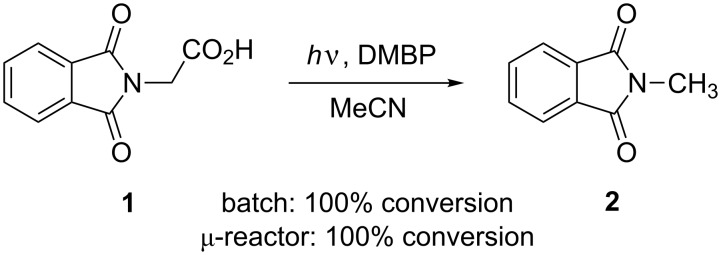
DMBP mediated α-photodecarboxylation of *N*-phthaloylglycine (**1**).

The photodecarboxylation of phthaloyl amino acids results in a formal exchange of –CO_2_H by –H and offers a convenient pathway to primary amines [40]. The reaction of *N*-phthaloylglycine (**1**) in acetonitrile using 0.1 equivalents of DMBP as a mediator was thus investigated as an early model transformation (Scheme 2). After 1 hour, complete conversions of **1** to *N*-methylphthalimide (**2**) were achieved in the batch and microreactor, as demonstrated by ^1^H NMR spectroscopy. In acetone-*d*_6_, the N–CH_3_ group in **2** showed a singlet at 3.11 ppm. DMBP remained unchanged and neither photoreduction nor photopinacolization products were detectable in the crude reaction mixture [41]. An attempt was made to isolate pure **2** by column chromatography but it eluted together with DMBP. The α-photodecarboxylation is, however, known to proceed with high selectivity [40].

### Photodecarboxylative cyclizations

**Scheme 3:**
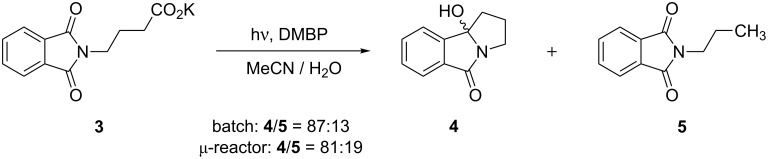
Photodecarboxylation of potassium phthaloyl-γ-aminobutyrate (**3**).

Two photodecarboxylative cyclization reactions were investigated with potassium phthaloyl-γ-aminobutyrate (**3**) and potassium phthalimidomethylsulfanylacetate (**6**) as starting materials [42]. A main advantage of these transformations is the ease of removal of the unreacted starting material by simple extraction. In contrast to acetone-sensitized reactions [43], DMBP-mediated irradiations of **3** furnished mixtures of the desired cyclization product **4** and the simple decarboxylation product **5** (Scheme 3; Table 1). When conducted in the batch system, **4** and **5** were obtained in a ratio of 87:13. Careful column chromatographic purification gave the polycyclic product **4** in an isolated yield of 29%. In the ^13^C NMR in acetone-*d*_6_, the C–OH group in **4** gave a characteristic singlet at 96.9 ppm. The simple decarboxylation product **5** eluted together with DMBP and could not be obtained in pure form. Its identity was thus confirmed by comparison with literature data. In acetone-*d*_6_, the terminal –CH_3_ group in **5** furnished a triplet at 0.90 ppm with a coupling constant of 7.4 Hz. Using the microreactor setup, a **4**/**5** mixture of 81:19 was isolated after 1 h of exposure. Despite the slightly lower selectivity, the cyclization product **4** was isolated in an improved yield of 47%. In both cases, DMBP showed no signs of decomposition suggesting that the reactions had not reached completion. Possible unreacted starting material was removed by extraction and no recovery attempts were made.

**Table 1:** Experimental results for the photodecarboxylation of **3**.

	batch	μ-reactor

time [h]	1	1
**4**/**5** ratio^a^	87/13	81/19
yield **4** [%]^b^	29	47

^a^Determined by ^1^H NMR analysis of the crude product. ^b^Isolated yield after column chromatography.

**Scheme 4:**
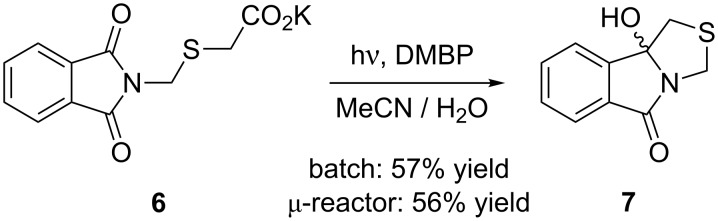
Photodecarboxylative cyclization of potassium phthalimidomethylsulfanylacetate (**6**).

When potassium phthalimidomethylsulfanylacetate (**6**) was used as the starting material, only the polycyclic thiazolidine derivative **7** could be isolated (Scheme 4). After purification by column chromatography, **7** was obtained in yields of 57% for the batch system and 56% for the microreactor. In acetone-*d*_6_, the methylene protons in the thiazolidine ring gave two sets of doublets at 2.97/3.39 ppm and 4.36/4.93 ppm. The increased yield of **7** compared to its carbon-analogue **3** suggests that the sulfur-atom in α-position to the carboxylate group in **6** accelerates photodecarboxylation [44–45]. In addition to the high chemoselectivity, DMBP remained photostable and could be reisolated almost quantitatively during chromatography.

### Photodecarboxylative additions

**Scheme 5:**
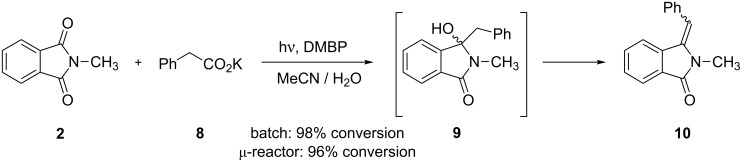
Photodecarboxylative benzylation of **2**.

Phthalimides can be efficiently alkylated by photodecarboxylation of carboxylates and this methodology has emerged as a powerful alternative to Grignard additions [46–48]. In contrast to the acetone-sensitized procedure, the DMBP mediated reaction of *N*-methylphthalimide (**2**) and potassium phenylacetate (**8**) furnished the corresponding phenylmethyleneisoindolinone **10** with high *E*-selectivity (Scheme 5; Table 2), as determined by comparison with literature data. In acetone-*d*_6_, the olefinic proton gave a clear singlet at 6.70 ppm. The formation of **10** can be explained by subsequent dehydration of the initially formed benzylated hydroxyphthalimidine **9**, a process favored by the extensive conjugation in **10** [49]. Under batch conditions, an almost complete conversion of **2** to **10** of 98% was achieved after 1 h of irradiation. Partial photoreduction of DMBP (ca. 10%) was furthermore observed. Using the same residence time, the transformation under microflow condition furnished a conversion to **10** of 96% but showed no decomposition of the mediator DMBP. Compound **10** could not be isolated in pure form but its NMR data was identical to that of an independently synthesized sample [49].

**Table 2:** Experimental results for the photobenzylation of **2**.

	batch	μ-reactor

time [h]	1	1
conversion [%]^a^	98	96
(*E*/*Z*)-**10** ratio^a^	>10/1	>10/1

^a^Determined by ^1^H NMR analysis of the crude product.

**Scheme 6:**
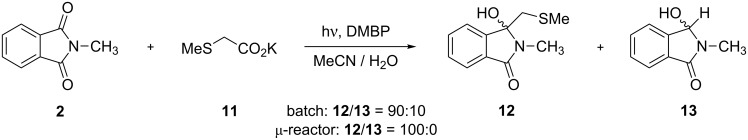
Photodecarboxylative addition of **11** to **2**.

Likewise, the addition of potassium 2-(methylthio)acetate (**11**) to *N*-methylphthalimide (**2**) was investigated [50]. In the larger Rayonet (batch) reactor, the photoreduction product of *N*-methylphthalimide, i.e., compound **13**, was identified next to the expected addition product **12** (Scheme 6; Table 3). In its ^1^H NMR spectrum in acetone-*d*_6_, the addition product **12** showed a pair of doublets for the –CH_2_S group at 3.20 and 3.27 ppm with a *^2^J* coupling constant of 14.0 Hz. In contrast, the reduction product **13** gave a doublet for its –CH group at 5.78 ppm, which changed into a singlet upon addition of D_2_O due to H/D exchange at the adjacent –OH group. Under batch conditions, the reaction had reached completion after 1 h and a 9:1 mixture of **12**/**13** was isolated. In addition, a large proportion of DMBP underwent photoreduction processes [41]. Column chromatography gave pure **12** and **13** in yields of 41% and 9%, respectively. When performed in the microreactor, the conversion was lower with 90%, but the transformation was highly selective. Neither the photoreduction product **13** nor any decomposition products of DMBP were identified by NMR analysis of the crude reaction mixture.

**Table 3:** Experimental results for the photodecarboxylative addition of **11** to **2**.

	batch	μ-reactor

time [h]	1	1
conversion [%]^a^	100	90
**12**/**13** ratio^a^	90/10	100/0
yield **12** [%]^b^	41	n.d.^c^

^a^Determined by ^1^H NMR analysis of the crude product. ^b^Isolated yield after column chromatography. ^c^Yield not determined.

**Scheme 7:**
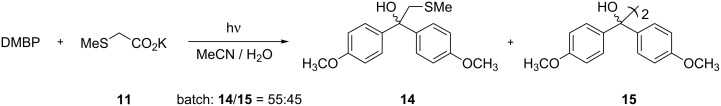
Photodecarboxylative addition of **11** to DMBP.

To investigate the role of DMBP, the reaction was repeated under batch conditions, but in the absence of *N*-methylphthalimide (**2**). After one hour, DMBP was completely consumed and its corresponding addition product **14** was obtained next to the expected benzpinacol **15** (Scheme 7). In acetone-*d*_6_, **14** gave singlets at 1.96 ppm for its –SCH_3_- and at 3.41 ppm for its –CH_2_S group, respectively. These values closely match those for the related benzophenone adduct [51]. The product ratio of **14**/**15** was determined to be 55:45.

### Reactor comparison

Based on the conversions or yields achieved, the two reactor systems showed very similar performances. Judged by the amounts of by-products, however, the product quality was somewhat superior for the microsystem. This finding is primarily attributed to the flow design of the dwell device which removes the product mixture from the irradiated area and consequently prevents follow-up reactions. The key parameters for the batch and microreactor are compiled in Table 4. Compared to the Schlenk tubes (50 mL and 100 mL), the irradiated area-to-volume (surface-to-volume) ratio of the dwell device was five to eight times larger with 1369 m^2^/m^3^. The dwell setup also gave the largest lamp power to irradiated area ratio of 1.74 W/cm^2^. The batch reactor incorporating the 50 mL Schlenk flask achieved a slightly lower value of 1.50 W/cm^2^, whereas the larger 100 mL Schlenk vessel gave the smallest ratio of 0.47 W/cm^2^.

**Table 4:** Technical details of the two reactor types.

Parameter	batch^a^	μ-reactor

aperture [cm^2^]^b^	85 / 274	86.1
irradiated area [cm^2^]	85 / 274	23.0
irradiated volume [cm^3^]	50 / 100	1.7
irradiated area/volume ratio [m^2^/m^3^]	171 / 274	1369
lamp power [W]	16 × 8	5 × 8
lamp power/aperture [W/cm^2^]	1.5 / 0.5	0.46
lamp power/irradiated area [W/cm^2^]	1.5 / 0.5	1.74

^a^Values given for 50 mL and 100 mL flask volumes. ^b^Assuming a cylindrical geometry for the Schlenk flask.

The conversion/yield per watt-hour (Wh), and the conversion/yield per Wh per irradiated area were furthermore determined for all transformations studied (Table 5) [38]. In all cases, the dwell device showed significantly larger energy efficiencies than the batch reactor. The values obtained for experiments with complete conversions represent the minimum energy efficiencies due to possible contributions from “over-irradiation”. Once all phthalimide is consumed, photoreduction of DMBP becomes the dominant reaction due to its continuing excitation [41]. The degree of these decomposition processes can thus be used as an indicator for “over-irradiation”.

**Table 5:** Energy efficiencies of the two reactor types.

Reaction	batch^a^		μ-reactor^a^	
	[% Wh^−1^]	[% Wh^−1^ cm^−2^]	[% Wh^−1^]	[% Wh^−1^ cm^−2^]

**1** → **2**	≥0.78^b^	≥0.0028^b^	≥2.5^b^	≥0.11^b^
**3** → **4**^c^	0.23	0.0027	1.18	0.05
**6** → **7**^c^	0.45	0.0052	1.40	0.06
**2** → **10**	0.77	0.0028	2.40	0.10
**2** → **12**	≥0.78^b^	≥0.0028^b^	2.25	0.09

^a^Batch: 128 Wh; microreactor: 40 Wh. ^b^Minimum values due to possible “over-irradiation”. ^c^Based on isolated yield of **4** or **7**.

### Mechanistic scenario

**Scheme 8:**
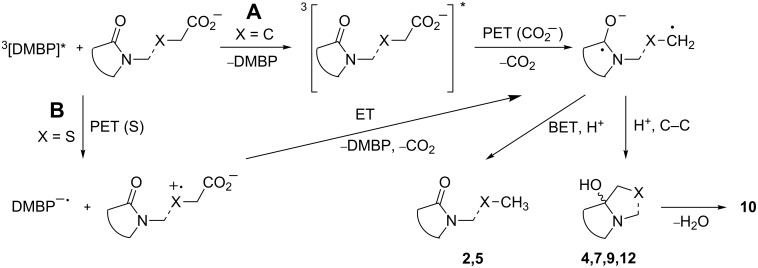
Mechanistic scenario (the broken line indicates intra- and intermolecular reactions).

For ketone-sensitized photodecarboxylations involving phthalimides, energy transfer and electron transfer processes have both been proposed [44,52]. A similar, simplified scenario is depicted in Scheme 8. Due to the comparable triplet energies of DMBP (*T*_1_ = 69.4 kcal/mol or 290 kJ/mol [53]) and phthalimides (**2**: *T*_1_ = 71 kcal/mol or 297 kJ/mol [39]), energy transfer (Scheme 8, path A) is not very efficient but has been confirmed spectroscopically for a related *N*-phthalimidocarboxylate/benzophenone pair [52]. Subsequent electron transfer (ET) from the carboxylate function to the triplet excited phthalimide furnishes an unstable carboxy radical, which undergoes rapid decarboxylation to the corresponding carbon radical. Protonation and C–C bond formation yields compounds **4** and **9**, and the latter undergoes further dehydration to **10**. Alternatively, back electron transfer (BET) and protonation generates the simple decarboxylation products **2** and **5**. Path A thus mirrors the mechanism proposed for acetone sensitization [43]. In contrast to carboxylates (for MeCO_2_^−^ calc. *E*_Ox_ = 1.54 V in MeCN versus SCE), thioethers (for Me_2_S: *E*_Ox_ = 1.23 V versus SCE) are more readily oxidized [23]. As a result, electron transfer to the triplet excited DMBP becomes energetically feasible (Scheme 8, path B) [52]. Similar electron transfer scenarios have been established for photoreactions of *N*-methylphthalimide or benzophenone with either thioethers [54–55] or alkyl- and arylthioacetic acids [48,56], respectively. With compound **6** or in the presence of **2**, successive electron transfer generates the corresponding phthalimide radical anion. Subsequent decarboxylation, protonation and C–C bond formation furnish products **7** and **12**. In the absence of *N*-methylphthalimide **2**, protonation and C–C bond formation to **14** or photopinacolization to **15** operate instead (not shown).

## Conclusion

DMBP mediated photodecarboxylation reactions involving phthalimides can be successfully transferred from batch to microflow conditions. While DMBP allows for the application of UVA light, its removal from the product remains challenging. Compared to their acetone-sensitized counterparts [27], however, selectivities and yields were reduced. We are therefore currently investigating water soluble or solid-supported photocatalysts that absorb in the UVA region. The results from this study nevertheless confirm the benefits of microflow reactors over batch systems in terms of energy efficiencies and selectivities. It is hoped that micro(flow)photochemistry will find future applications in chemical and pharmaceutical R&D processes [14,57].

## Supporting Information

Supporting Information contains full experimental procedures and NMR data of all photoproducts.

File 1 Full experimental details and NMR data.
