# Supplementary material for: Microphotochemistry: 4,4'-Dimethoxybenzophenone mediated photodecarboxylation reactions involving phthalimides
Source: Beilstein J Org Chem. 2011 Aug 2;7:1055–63. doi: 10.3762/bjoc.7.121 (PMC3170193; doi:10.3762/bjoc.7.121)
Supplement: File 1 — Full experimental details and NMR data. [file Beilstein_J_Org_Chem-07-1055-s001.pdf]

Supporting Information

for

Microphotochemistry: 4,4'-Dimethoxybenzophenone mediated photodecarboxylation reactions involving phthalimides

Oksana Shvydkiv[1], Kieran Nolan[1] and Michael Oelgemöller*[2]

Address: [1]School of Chemical Sciences, Dublin City University, Dublin 9, Ireland and [2]School of Pharmacy and Molecular Sciences, James Cook University, Townsville, QLD 4811, Australia.

Email: Michael Oelgemöller* - michael.oelgemoeller@jcu.edu.au

*Corresponding author

Full experimental details and NMR data

Experimental

General

All commercially available starting materials and reagents were purchased from Sigma-Aldrich and were used without further purification. The syntheses of compounds **3** and **6** have been described previously [1,2]. NMR spectra were recorded on a Bruker 400 Ultrashield™ (^{1}H: 400 MHz and ^{13}C: 100 MHz) using the residual solvent signal as internal standard. Chemical shifts (δ) are given in ppm; coupling constants (*J*) in Hz. Melting points were determined using a Griffin Melting Point Apparatus and are uncorrected. Preparative chromatography was carried out using Merck silica gel 60 and ethyl acetate/*n*-hexane (1:1). Batch irradiations were conducted in a Rayonet RPR-200 chamber reactor (Southern New England) equipped with 16 × RPR-3500Å lamps (16 × 8 W) and using Pyrex flasks. Microflow reactions were performed in a dwell device (mikroglas chemtech) under a Luzchem UV exposure panel equipped with 5 × RPR-3500Å lamps (5 × 8 W). All products are literature known.

Irradiations

α-Photodecarboxylation of phthaloylglycine 1 under batch conditions: A solution of **1** (1.5 mmol) and DMBP (0.15 mmol) in acetonitrile (100 mL) was irradiated for 1 h while a gentle stream of nitrogen was flushed through the solution. After evaporation of the solvent, the conversion was determined by ^{1}H NMR spectroscopy of the crude product. The signal integration for the $NC\underline{H}_2$ protons of **1** was compared with the signal integration for the $NC\underline{H}_3$ protons of **2**. For characterization purposes, a sample of **2** was furthermore isolated by column chromatography.

α-Photodecarboxylation of phthaloylglycine 1 under microflow conditions: A solution of **1** (0.225 mmol) and DMBP (22.5 µmol) in acetonitrile (15 mL) was degassed with nitrogen and loaded into a syringe pump. The reaction mixture was pumped through the dwell device (residence time of 1 h) and was irradiated with UVA light. After evaporation of the solvent, the conversion rate was established by ^{1}H NMR analysis.

***N*-Methylphthalimide (2).** Colorless needles; R_f: 0.62 (ethyl acetate/*n*-hexane 1:1); mp 133–134ºC (lit. [3]: mp 135 °C); ^{1}H NMR (400 MHz, acetone-d_6) δ 3.11 (s, 3H, NCH_3), 7.83 (m, 4H, H_{arom}) ppm; ^{13}C NMR (100 MHz, acetone-d_6) δ 23.9, 123.6, 133.3, 134.8, 168.8 ppm.

Photodecarboxylative cyclization of 3 and 6 under batch conditions: A solution of **3** or **6** (0.75 mmol) and DMBP (75 µmol) in an acetonitrile/water mixture (1:1 vol %, 50 mL) was irradiated for 1 h while being purged with nitrogen. Most of the acetonitrile was evaporated and the crude product mixture was extracted with CH_2Cl_2 (3 × 30 mL). The combined organic layer was washed with sat. $NaHCO_3$ (2 × 30 mL), brine (2 × 30 mL) and dried over $MgSO_4$. After filtration and evaporation of the solvent, the crude products were analyzed by ^{1}H NMR spectroscopy. The **4**/**5** ratio was determined by integration and comparison of a $NC\underline{H}_2$ proton of **4** with the terminal $C\underline{H}_3$ protons of **5**. Pure **4** and **7** were isolated by column chromatography.

Photodecarboxylative cyclization of 3 and 6 under microflow conditions: A solution of **3** or **6** (0.225 mmol) and DMBP (22.5 µmol) in an acetonitrile/water mixture (1:1 vol %, 15 mL) was purged with nitrogen and loaded into a syringe pump. The reaction mixture was pumped through the microreactor (residence time of 1 h) while being exposed to UVA light. The work-up, product analysis and purification steps were the same as for the batch system.

9b-Hydroxy-1,2,3,9b-tetrahydro-5H-pyrrolo[2,1-a]isoindol-5-one (4). Colorless solid; R_f: 0.30; mp 125–127 °C (lit. [4]: mp 123–125 °C); ^{1}H NMR (400 MHz, acetone-d_6) δ 1.50 (m, 1H, CH_2), 2.24 (m, 2H, CH_2), 2.53 (m, 1H, CH_2), 3.32 (m, 1H, NCH_2), 3.57 (m, 1H, NCH_2), 5.22 (s, 1H, OH), 7.48 (m, 1H, H_{arom}), 7.57 (m, 3H, H_{arom}) ppm; ^{13}C NMR (100 MHz, acetone-d_6) δ 28.3, 36.0, 42.1, 96.9, 123.5, 123.7, 130.1, 133.0, 133.2, 149.2, 170.1 ppm.

***N*-Propylphthalimide (5).** R_f: 0.68; ^{1}H NMR (400 MHz, acetone-d_6) δ 0.90 (t, ^3J = 7.4 Hz, 3H, CH_3), 1.65 (q, ^3J = 7.4 Hz, 2H, CH_2), 3.59 (t, ^3J = 7.4 Hz, 2H, NCH_2), 7.84 (m, 4H, H_{arom}) ppm.

9b-Hydroxy-1,9b-dihydrothiazolo[4,3-a]isoindol-5-one (7). Colorless solid; R_f: 0.31; mp 121–122 °C (lit [5]: 124–125 °C); ^{1}H NMR (400 MHz, acetone-d$_6$) δ 2.97 (d, ^2J = 11.2 Hz, 1H, CH_2S), 3.39 (d, ^2J = 11.2 Hz, 1H, CH_2S), 4.36 (d, ^2J = 8.8 Hz, 1H, NCH_2S), 4.93 (d, ^2J = 8.8 Hz, 1H, NCH_2S), 5.83 (s, 1H, OH), 7.57 (m, 1H, H_{arom}), 7.64 (dd, ^3J = 7.6 Hz, ^4J = 1.2 Hz, 1H, H_{arom}), 7.71 (m, 2H, H_{arom}) ppm; ^{13}C NMR (100 MHz, acetone-d_6) δ 40.2, 42.7, 98.4, 123.4, 123.9, 130.8, 131.3, 134.1, 147.6, 167.9 ppm.

Photodecarboxylative additions of 8 and 11 to 2 under batch conditions: A solution of **2** (1.5 mmol), DMBP (0.15 mmol) and **8** or **11** (4.5 mmol) in an acetonitrile/water mixture (1:1 vol %, 100 mL) was irradiated for 1 h while being degassed with nitrogen. Most of the acetonitrile was evaporated and the crude product mixture was extracted with CH_2Cl_2 (3 × 50 mL). The combined organic layer was washed with sat. $NaHCO_3$ (2 × 50 mL), brine (2 × 50 mL), dried over $MgSO_4$, filtered and evaporated. The composition was determined by ^{1}H NMR spectroscopy of the crude product. Conversions were established by comparing the NMR integration values of the $NC\underline{H}_3$ signals. The ratio of **12**/**13** was calculated by

comparing the integration of a C<u>H</u>$_2$S proton of **12** with the C<u>H</u>N proton of **13**. Pure **12** or **13** were isolated by column chromatography.

Photodecarboxylative additions of 8 and 11 to 2 under microflow conditions: A solution of **2** (0.225 mmol), DMBP (22.5 µmol) and **8** or **11** (0.675 mmol) in an acetonitrile/water mixture (1:1 vol %, 15 mL) was purged with nitrogen and loaded into a syringe pump. The reaction mixture was pumped through the microreactor while being exposed to light for 1 h. The workup procedure and product analysis were the same as for the batch system.

(3*E*)–3–Benzylidene–2–methylisoindolin–1–one (10). R_f: 0.55; ^{1}H NMR (400 MHz, acetone-d_6): δ 3.36 (s, 3H, NCH_3), 6.69 (s, 1H, H_{olef}), 7.35 (d, ^3J = 7.6 Hz, 1H, H_{arom}), 7.41 (m, 2H, H_{arom}), 7.48 (m, 5H, H_{arom}), 7.75 (d, ^3J = 7.6 Hz, 1H, H_{arom}) ppm; ^{13}C NMR (100 MHz, $CDCl_3$) δ 26.3, 110.4, 123.1, 127.9, 128.8, 129.3, 129.7, 130.8, 131.6, 134.0, 135.0, 135.4, 137.6, 166.7 ppm.

3-Hydroxy-2-methyl-3-(methylthiomethyl)isoindolin-1-one (12). Colorless solid; R_f: 0.26; mp 110–114 °C (lit. [5]: 114–116 °C); ^{1}H NMR (400 MHz, acetone-d_6): δ 1.76 (s, 3H, SCH_3), 2.92 (s, 3H, NCH_3), 3.20 (d, ^2J = 14.0 Hz, 1H, CH_2), 3.27 (d, ^2J = 14.0 Hz, 1H, CH_2), 5.47 (s, 1H, OH), 7.50 (ddd, ^3J = 7.6, ^3J = 7.6, ^4J = 1.2 Hz, 1H, H_{arom}), 7.58 (ddd, ^3J = 7.6, ^3J = 7.6, ^4J = 1.2 Hz, 1H, H_{arom}), 7.64 (dd, ^3J = 7.6, ^4J = 1.2 Hz, 1H, H_{arom}), 7.71 (dd, ^3J = 7.6, ^4J = 1.2 Hz, 1H, H_{arom}) ppm; ^{13}C NMR (100 MHz, acetone-d_6): δ 16.7, 23.5, 40.8, 90.9, 123.1, 123.3, 130.2, 132.5, 133.1, 147.8, 167.1 pm.

3-Hydroxy-2-methyl-1,3-dihydroisoindol-1-one (13). Colorless solid; R_f: 0.16; mp 127–129 °C (lit. [6]: 128–129 °C); ^{1}H NMR (400 MHz, $CDCl_3$) δ 3.04 (s, 3H, NCH_3), 5.40 (d, ^3J = 9.6 Hz, 1H, OH), 5.78 (d, ^3J = 9.6 Hz, 1H, CHN), 7.50 (m, 1H, H_{arom}), 7.60 (m, 2H, H_{arom}), 7.65 (d, ^3J = 7.6 Hz, 1H, H_{arom}) ppm; ^{13}C NMR (100 MHz, $CDCl_3$) δ 26.0, 83.5, 122.8, 123.1, 131.2, 132.0, 133.8, 143.8, 167.6 pm.

Photodecarboxylative addition of 11 to DMBP under batch conditions: A solution of DMBP (0.075 mmol) and **11** (2.25 mmol) in an acetonitrile/water mixture (1:1 vol %, 50 mL) was irradiated for 1 hour while a gentle stream of nitrogen was passed through the solution. Most of the acetonitrile was evaporated and the crude product mixture was extracted with CH_2Cl_2 (3 × 50 mL). The combined organic layer was washed with sat. $NaHCO_3$ (2 × 50 mL), brine (2 × 50 mL), dried over $MgSO_4$, filtered and evaporated. The crude product was analyzed by ^{1}H NMR spectroscopy. The ratio of **14**/**15** was calculated by comparing integrations for $C\underline{H}_{arom}$ protons.

1,1-Bis(4-methoxyphenyl)-2-(methylthio)ethanol (14). ^{1}H NMR (400 MHz, acetone-d_6) δ 1.96 (s, 3H, SCH_3), 3.41 (s, 2H, CH_2S), 3.76 (s, 6H, OCH_3), 4.38 (s, 1H, OH), 6.84 (d, ^3J = 9.2 Hz, 4H, H_{arom}), 7.39 (d, ^3J = 9.2 Hz, 4H, H_{arom}) ppm.

1,1,2,2-Tetrakis(4-methoxyphenyl)ethane-1,2-diol (15). ^{1}H NMR (400 MHz, acetone-d_6) δ 3.72 (s, 12H, OCH_3), 4.47 (s, 2H, OH), 6.65 (d, ^3J = 9.2 Hz, 8H, H_{arom}), 7.31 (d, ^3J = 9.2 Hz, 8H, H_{arom}) ppm.

References

1. Griesbeck, A. G.; Henz, A.; Kramer, W.; Lex, J.; Nerowski, F.; Oelgemöller, M.; Peters, K.; Peters, E.-M. *Helv. Chim. Acta*, **1997**, *80*, 912–933. doi:10.1002/hlca.19970800324
2. Griesbeck, A. G.; Oelgemöller, M.; Lex, J.; Haeuseler, A.; Schmittel, M. *Eur. J. Org. Chem.*, **2001**, 1831–1843. doi:10.1002/1099-0690(200105)2001:10<1831::AID-EJOC1831>3.0.CO;2-7
3. Schindlbauer, H. *Monatsh. Chem.* **1973**, *104*, 848–853. doi:10.1007/BF00910596

4. Lee, Y. J.; Ling, R.; Mariano, P. S.; Yoon, U. C.; Kim, D. U.; Oh, S. W. *J. Org. Chem.*, **1996**, *61*, 3304–3314. doi:10.1021/jo9522623
5. Hatanaka, Y.; Sato, Y.; Nakai, H.; Wada, M.; Mizuguchi, T.; Kanaoka, Y. *Liebigs Ann. Chem*., **1992**, 1113–1123. doi:10.1002/jlac.1992199201184
6. Ress, C. W.; Sabet, C. R. *J. Chem. Soc.* **1965**, 687–691.
